# Comparison of efficacy and safety among axitinib, sunitinib, and sorafenib as neoadjuvant therapy for renal cell carcinoma: a retrospective study

**DOI:** 10.1186/s40880-019-0405-5

**Published:** 2019-10-11

**Authors:** Wen Cai, Biao Cai, Juan Zhou, Yonghui Chen, Jin Zhang, Yiran Huang, Wei Xue, Jiwei Huang

**Affiliations:** 1 Department of Urology, Renji Hospital, School of Medicine, Shanghai Jiao Tong University, 160 Pujian Rd., Pudong District, Shanghai, 200127 P. R. China; 2 Bio-X Institutes, Key Laboratory for the Genetics of Developmental and Neuropsychiatric Disorders (Ministry of Education), Shanghai Jiao Tong University, Shanghai, 200030 P. R. China

Dear editor,

Renal cancer accounts for approximately 2% of all cancer deaths worldwide, and renal cell carcinoma (RCC) is the predominant subtype [1]. Radiotherapy and chemotherapy have been found to have limited roles in the treatment of RCC. However, the treatment outcomes of metastatic RCC (mRCC) have been improved drastically since the application of molecular targeted therapeutic agents such as tyrosine kinase inhibitors (TKIs). TKIs have been gradually used for the preoperative treatment of RCC.

Neoadjuvant therapy of advanced RCC mainly aims to prevent local progression and possible metastasis, increasing the feasibility of tumor resection for certain patients. After applying targeted therapy before surgery, primary and metastatic tumor shrinkage or stabilization could be achieved in 70%–80% of mRCC patients [2]. Sunitinib was the first targeted agent to be used as neoadjuvant therapy for RCC, reported in 2008 [3]. Subsequently, several trials were conducted to study the efficacy of sunitinib as neoadjuvant therapy, after which the rate of primary tumor shrinkage was reported to be about 15%–20% [4, 5]. Nowadays, several TKIs, such as sorafenib [6] and axitinib [7], have been used as neoadjuvant therapy for RCC. However, Chinese-specific safety and efficacy data comparing these targeted agents are currently lacking, which would be a critical basis for drug selection in neoadjuvant therapy of RCC. Hence, here we report the results of a single-institution clinical trial that was designed to evaluate and compare the efficacy and safety data of axitinib, sunitinib, and sorafenib as neoadjuvant therapy for RCC in Chinese patients.

The methods in the present study are detailed in Additional file 1: Patients and methods. The clinicopathological characteristics of 69 RCC patients are shown in Additional file 2: Table S1. Of them, 44 (63.8%) were males, and 25 (36.5%) were females. Their median age was 58 years (interquartile range 31–76 years). The majority of patients had Fuhrman Grade 3 RCC (50.7%) and had stage T3 disease (44.9%). Fifteen (21.7%) patients were treated with axitinib, 24 (34.8%) with sunitinib, and 30 (43.5%) with sorafenib. No significant difference in clinicopathological characteristics among these three groups was observed.

At 12 weeks after neoadjuvant therapy initiation, changes in the maximal diameter of the primary tumor were investigated, as demonstrated in Fig. 1, and tumor responses were recorded (Table 1). The median reduction of maximal tumor diameter was 1.5 cm in the axitinib group, which was significantly higher than that in the sunitinib group (0.8 cm, *P* = 0.001) and the sorafenib group (0.5 cm, *P* < 0.001). The median reduction rate was also significantly higher in the axitinib group than in the sunitinib and sorafenib groups (both *P* = 0.001). In the axitinib group, 2 (13.3%) patients were evaluated as having partial response (PR), and 13 (86.7%) had stable disease (SD); 13 (86.7%) had > 10% reduction in maximal tumor diameter; 2 (13.3%) achieved tumor downstaging (1 from T2a to T1b and 1 from T1b to T1a). In the sunitinib group, 23 (95.8%) patients had SD, and 1 (4.2%) had progressive disease (PD); 16 (66.7%) had > 10% reduction in maximal tumor diameter; 1 (4.2%) achieved tumor downstaging (from T1b to T1a). In the sorafenib group, 1 (3.3%) patient had PR, and 29 (96.7%) had SD; 8 (26.7%) had > 10% reduction in maximal tumor diameter; 2 (6.7%) achieved tumor downstaging (both from T1b to T1a).

**Fig. 1:**
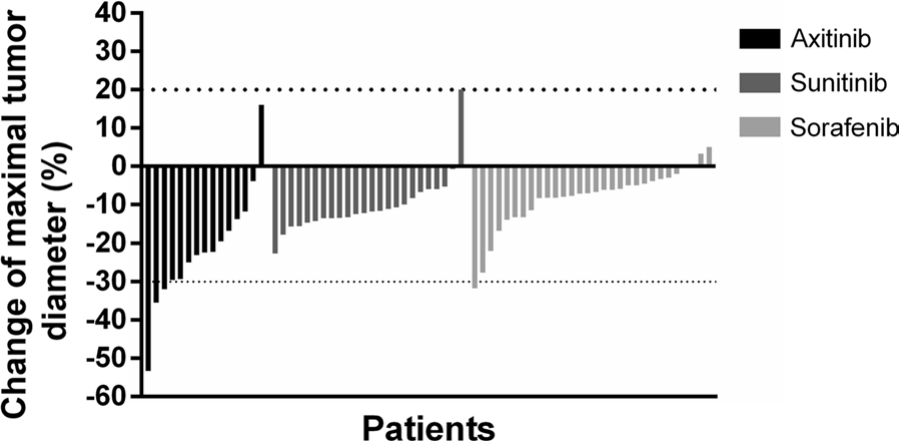
Waterfall plot of the change of maximal tumor diameter at 12 weeks after neoadjuvant therapy initiation. Each bar represents the datum of 1 individual patient. Negative values represent diameter reduction, and positive values represent diameter increment. A change greater than − 30% indicates partial response, − 30% to 20% indicates stable disease, and greater than 20% indicates progressive disease

**Table 1:** Changes of primary tumor in patients with renal cell carcinoma before and at 12 weeks after neoadjuvant therapy

Variable	Axitinib	Sunitinib	Sorafenib	*P* ^1^	*P* ^2^	*P* ^3^
Maximal diameter (cm)^a^						
Before therapy	7.2 (6.2–8.5)	6.4 (5.7–10.3)	6.8 (5.8–11.0)	0.497	0.664	0.868
After therapy	5.2 (4.4–7.4)	5.6 (5.1–9.8)	6.4 (4.8–6.4)	0.266	0.284	0.657
Reduction	1.5 (1.2–2.2)	0.8 (0.5–1.1)	0.5 (0.4–0.8)	0.001	< 0.001	0.024
Reduction rate (%)^a^	22.4 (13.8–30)	12.2 (7.1–14.1)	6.9 (3.7–12.0)	0.001	0.001	0.023
> 10% reduction [cases (%)]	13 (86.7)	16 (66.7)	8 (26.7)	0.310*	0.001*	0.003*

*P*^1^, axitinib vs. sunitinib; *P*^2^, axitinib vs. sorafenib; *P*^3^, sunitinib vs. sorafenib

*P*, Wilcoxon signed-rank test; P*, Pearson’s Chi square test

^a^The data are presented as median (interquartile range)

Adverse events (AEs) are summarized in Table 2. Grade 3–4 AEs were recorded in 2 (13.3%) patients in the axitinib group, 9 (37.5%) in the sunitinib group, and 6 (20.0%) in the sorafenib group. Nine (37.5%) patients in the sunitinib group received AE-related dose reduction (to 37.5 mg once daily). The AEs which were significantly more common in the sunitinib group than in the axitinib group included leukocytopenia (45.9% vs. 13.3%, *P* = 0.036) and thrombocytopenia (50.0% vs. 23.3%, *P* = 0.041).

**Table 2:** Adverse events associated with different neoadjuvant therapy in patients with renal cell carcinoma

Adverse event	Axitinib [cases (%)]			Sunitinib [cases (%)]			Sorafenib [cases (%)]			*P* ^1^	*P* ^2^	*P* ^3^
	All grade	Grade 1–2	Grade 3–4	All grade	Grade 1–2	Grade 3–4	All grade	Grade 1–2	Grade 3–4			
Hand–foot syndrome	6 (40.0)	5 (33.3)	1 (6.7)	13 (54.2)	10 (41.7)	3 (12.5)	16 (53.3)	13 (43.3)	3 (10.0)	0.389	0.399	0.951
Hypertension	6 (40.0)	5 (33.3)	1 (6.7)	11 (45.9)	8 (33.3)	3 (12.5)	13 (43.3)	10 (33.3)	2 (6.7)	0.721	0.831	0.854
Diarrhea	4 (29.4)	4 (29.4)	0 (0.0)	9 (37.5)	9 (37.5)	0 (0.0)	12 (40.0)	11 (36.7)	1 (3.3)	0.485	0.378	0.852
Nausea	4 (26.7)	4 (26.7)	0 (0.0)	8 (33.3)	8 (33.3)	0 (0.0)	8 (26.7)	8 (26.7)	0 (0.0)	0.092	1.000	0.594
Fatigue	4 (26.7)	4 (26.7)	0 (0.0)	7 (29.1)	7 (29.1)	0 (0.0)	8 (26.7)	8 (26.7)	0 (0.0)	0.150	1.000	0.839
Loss of appetite	3 (20.0)	3 (20.0)	0 (0.0)	6 (25.0)	6 (25.0)	0 (0.0)	7 (23.3)	7 (23.3)	0 (0.0)	0.718	0.800	0.887
Leukocytopenia	2 (13.3)	2 (13.3)	0 (0.0)	11 (45.9)	8 (33.3)	3 (12.5)	8 (26.7)	7 (23.3)	1 (3.3)	0.036	0.526	0.143
Thrombocytopenia	2 (13.3)	2 (13.3)	0 (0.0)	12 (50.0)	9 (37.5)	3 (12.5)	7 (23.3)	7 (23.3)	0 (0.0)	0.020	0.693	0.041
lymphocytopenia	2 (13.3)	3 (13.3)	0 (0.0)	8 (33.3)	5 (20.8)	3 (12.5)	4 (13.3)	4 (13.3)	0 (0.0)	0.310	1.000	0.079
Anemia	2 (13.3)	2 (13.3)	0 (0.0)	5 (20.8)	4 (15.8)	1 (4.2)	7 (23.3)	6 (20.0)	1 (3.3)	0.638	0.890	0.826
Hypothyroidism	2 (13.3)	2 (13.3)	0 (0.0)	6 (25.0)	8 (25.0)	0 (0.0)	4 (13.33)	4 (13.33)	0 (0.0)	0.638	1.000	0.457

*P*^1^, axitinib vs. sunitinib; *P*^2^, axitinib vs. sorafenib; *P*^3^, sunitinib vs. sorafenib

Although no direct study showed that neoadjuvant targeted therapy could prolong overall survival, it has been proven to be able to make some unresectable renal tumors resectable or make partial nephrectomy possible for some complex renal tumors [8]. Generally, the degree of primary tumor reduction represents the principle assessment criterion for neoadjuvant therapy. Abel et al. [9] retrospectively analyzed the responses of primary renal tumors to 6 types of neoadjuvant targeted therapy among 168 patients with mRCC. They reported that only 5.9% of their patients had > 30% primary tumor reduction, suggesting that neoadjuvant targeted therapy might only benefit certain patients. However, Karam et al. [7] reported that almost half of the patients (45.8%) who received axitinib neoadjuvant therapy achieved > 30% primary tumor reduction and 54.2% were evaluated as having SD. A study by Karam et al. [7] also demonstrated the efficacy of axitinib as neoadjuvant therapy for RCC, but no direct comparison with other drugs was made. Thus, the superiority of axitinib over other targeted agents is still questionable.

In the present study, median tumor reduction rates were 12.2% and 6.9% in the sunitinib and sorafenib groups, respectively, with 1 PR observed in the sorafenib group and none in the sunitinib group. In the axitinib group, 2 patients achieved > 30% reduction, and the overall reduction rate (22.4%) was significantly higher than those in the sunitinib (*P* = 0.001) and sorafenib groups (*P* = 0.023). The degree of tumor shrinkage in the sunitinib group was lower than those reported in literature [4, 5]. A possible explanation could be that Chinese patients have lower tolerance to sunitinib, compared with the western population. In the present study, 9 (37.5%) patients received dose reduction or interruption due to intolerable AEs. As reported in previous studies, the efficacy of low-dose targeted agents was inferior to that of high-dose agents [3, 4].

TKIs inhibit tumor growth by tumor angiogenesis inhibition and they might also influence normal vessels and increase the risk of incision bleeding after nephrectomy surgery. Thus, postoperative bleeding and incision healing are important criteria for the safety evluation of neoadjuvant therapy. Axitinib has a half-life of 2.5–6.1 h. Therefore, withdrawing axitinib treatment 1–2 weeks before surgery would not increase the risk of postoperative bleeding. Similarly, the half-life of sunitinib is 41–86 h, and that of sorafenib is 25–48 h. They should be withdrawn 3–4 weeks before surgery. Chapin et al. [10] have evaluated the effects of neoadjuvant targeted therapy on the risk of postoperative complications in mRCC patients. The rate of incision complications in their report was higher than that in the present study, possibly due to larger amount of stage T3–4 patients in their study. Chapin et al. [10] and our team have confirmed the safety of neoadjuvant targeted therapy, proving that the risk of serious postoperative complications would not be increased for RCC patients.

The AE profile is another crucial aspect for drug selection and could vary to a great degree for different individuals. Sunitinib is more likely to induce myelosuppression in Asian patients compared with Westerners [11]. AE-related dose reduction or interruption could reduce the overall efficacy of targeted agents. In the present study, the rates of leukocytopenia and thrombocytopenia were higher in the sunitinib group than in the axitinib group, leading to a higher rate of AE-related dose reduction or interruption in the sunitinib group.

The limitations of the present study are as follows. First, this was a retrospective single-institutional study which may contain inevitable selection bias. Second, the sample size was small; however, we believe these significant findings could serve as important guidelines for neoadjuvant therapy of RCC. Last, there was no clear standard for drug selection. Thus, long-term prospective studies are warranted to further compare the efficacy and safety of different targeted agents as neoadjuvant therapy for RCC.

To conclude, our findings suggest that compared with sunitinib and sorafenib, axitinib could significantly increase tumor reduction with better patient tolerance. Therefore, axitinib might be a superior targeted agent for neoadjuvant therapy in the treatment of RCC. Randomized controlled trials of larger-scale are recommended to further compare the efficacy of different targeted drugs. Patients could also be further stratified to obtain a more detailed efficacy and safety profile and to establish a more accurate drug selection guideline.

## Supplementary information

**Additional file 1.** Patients and methods.

**Additional file 2: Table S1.** Clinicopathological characteristics and tumor responses of patients with renal cell carcinoma receiving neoadjuvant therapy.

## Data Availability

Not applicable.

## Abbreviations

AE: adverse events
mRCC: metastatic renal cell carcinoma
RCC: renal cell carcinoma
TKI: tyrosine kinase inhibitor
