# Supplementary material for: Comparison of efficacy and safety among axitinib, sunitinib, and sorafenib as neoadjuvant therapy for renal cell carcinoma: a retrospective study
Source: Cancer Commun (Lond). 2019 Oct 11;39:56. doi: 10.1186/s40880-019-0405-5 (PMC6788019; doi:10.1186/s40880-019-0405-5)
Supplement: Supplementary file 1 — Additional file 1. Patients and methods. [file 40880_2019_405_MOESM1_ESM.docx]

**Patients and Methods**

***Study design and population***

Data were retrieved from the electronic medical records and laboratory reports of RCC patients who visited the department of Urology in Renji Hospital affiliated to Shanghai Jiao Tong University School of Medicine between January 2010 and December 2017. This study involved patients who received axitinib, sunitinib, or sorafenib as neoadjuvant therapy before nephrectomy. The goals of neoadjuvant therapy were to make a partial nephrectomy feasible, downsize the tumor thrombus, or make unresectable tumor resectable.

***Treatment and follow-up***

Axitinib was administered 5 mg twice daily orally for 8-12 weeks. Sunitinib was prescribed 50 mg once daily orally (on a 4-week-on/2-week-off schedule) for 8-12 weeks. Sorafenib was administered 400 mg twice daily orally for 8-12 weeks. Dose titrations were done considering the patient’s tolerance. Axitinib was reduced to 3 mg twice daily, sunitinib was reduced to 37.5 mg once daily, and sorafenib was reduced to 200 mg twice daily. Surgery was performed 1-2 weeks after the completion of axitinib treatment or 3-4 weeks after that of sorafenib or sunitinib treatment.

***Outcome measures***

Fuhrman’s grade and clinical stage were based on the examination of nephrectomy specimens. According to the 2009 TNM classification of the [American Joint Committee on Cancer](http://www.baidu.com/link?url=MfzHJidDLKmdasy6KgonSVeAVsmCMcOVOf4uvuY4-pVU7klmAarFgnt5_lqeo99P) (AJCC) ^[1]^. Clinical response of the primary tumor after neoadjuvant therapy was assessed according to Response Evaluation Criteria in Solid Tumors V1.1 ^[2]^. National Cancer Institute Common Terminology Criteria for Adverse Events version V4.0 ^[3]^ were used for grading of treatment-related adverse events (AEs). The primary outcome was the change of maximal tumor diameter. The secondary outcome was AEs. All patients were suggested to have imaging examinations before neoadjuvant therapy and 8-12 weeks after neoadjuvant therapy initiation. Computed tomography or magnetic resonance imaging was used to evaluate primary tumor. The imaging assessments of the same patient were performed by the same doctor.

***Statistical analyses***

All analyses were performed using the SAS version 9.0 (SAS Corporation, Cary, NC, USA). Wilcoxon signed rank test was used to compare tumor diameter change and the reduction rate of tumor diameter among the axitinib, sunitinib, and sorafenib groups. The categorical variables such as clinicopathological characteristics, tumor responses, and AEs are presented as number of patients and were analyzed using Pearson’s Chi-square test. *P* < 0.05 was considered statistically significant.

References

[1] Edge, S.B., Compton, C.C. The American Joint Committee on Cancer: the 7th edition of the AJCC cancer staging manual and the future of TNM. Ann Surg Oncol. 2010 Jun;17(6):1471-4.

[2] Duffaud F, Therasse P. New guidelines to evaluate the response to treatment in solid tumors. Bull Cancer. 2001;87:881–6.

[3] Common Terminology Criteria for Adverse Events v4.0 (CTCAE). Washington, DC: US Department of Health and Human Services. https://evs.nci.nih.gov/ftp1/CTCAE/CTCAE_4.03/CTCAE_4.03_2010-06-14_QuickReference_5x7.pdf.
