# Supplementary material for: Comparison of efficacy and safety among axitinib, sunitinib, and sorafenib as neoadjuvant therapy for renal cell carcinoma: a retrospective study
Source: Cancer Commun (Lond). 2019 Oct 11;39:56. doi: 10.1186/s40880-019-0405-5 (PMC6788019; doi:10.1186/s40880-019-0405-5)
Supplement: Supplementary file 2 — Additional file 2: Table S1. Clinicopathological characteristics and tumor responses of patients with renal cell carcinoma receiving neoadjuvant therapy. [file 40880_2019_405_MOESM2_ESM.docx]

**Table S1. Clinicopathological characteristics and tumor responses of patients with renal cell carcinoma receiving neoadjuvant therapy**

| **Variable** | **Total [cases (%)]** | **Axitinib [cases (%)]** | **Sunitinib [cases (%)]** | **Sorafenib [cases (%)]** | **𝑃 value** |
| --- | --- | --- | --- | --- | --- |
| **Total** | **69** | **15** | **24** | **30** |  |
| Sex |  |  |  |  | 0.288 |
| Male | 44 (63.8) | 11 (73.3) | 17 (70.8) | 16 (53.3) |  |
| Female | 25 (36.2) | 4 (26.7) | 7 (29.2) | 14(46.7) |  |
| Age |  |  |  |  | 0.997 |
| < 65 years | 55 (79.7) | 12(80.0) | 19 (79.2) | 24 (80.0) |  |
| ≥ 65 years | 14 (20.3) | 3 (20.0) | 5 (20.8) | 6 (20.0) |  |
| Tumor side |  |  |  |  | 0.689 |
| Left kidney | 29 (42.0) | 7 (46.7) | 8 (33.3) | 14 (46.7) |  |
| Right kidney | 34 (49.3) | 6 (40.0) | 14 (58.3) | 14 (46.7) |  |
| Bilateral kidneys | 6 (8.7) | 2 (13.3) | 2 (8.3) | 2 (6.7) |  |
| Histological type |  |  |  |  | 0.969 |
| Clear cell RCC | 64 (92.8) | 14 (93.3) | 22 (91.7) | 28 (93.3) |  |
| Papillary RCC | 5 (7.2) | 1 (6.7) | 2 (8.3) | 2 (6.7) |  |
| Fuhrman grade |  |  |  |  | 0.994 |
| 2 | 31 (44.9) | 6 (40.0) | 11 (45.8) | 14 (46.7) |  |
| 3 | 35 (50.7) | 9 (60.0) | 12 (50.0) | 14 (46.7) |  |
| 4 | 3 (4.3) | 0 (0.0) | 1 (4.2) | 2 (6.7) |  |
| Nodal status |  |  |  |  | 0.668 |
| N0 | 54 (78.3) | 13 (86.7) | 18 (75.0) | 23 (76.7) |  |
| N1 | 15 (21.7) | 2 (13.3) | 6 (25.0) | 7 (23.3) |  |
| Pretreatment T stage |  |  |  |  | 0.384 |
| T1b | 23 (33.3) | 5 (33.3) | 7 (29.2) | 11 (36.7) |  |
| T2 | 11 (15.9) | 6 (40.0) | 2 (8.3) | 3 (10.0) |  |
| T3 | 31 (44.9) | 4 (26.7) | 14 (58.3) | 13 (43.3) |  |
| T4 | 4 (5.8) | 0 (0.0) | 1 (4.2) | 3 (10.0) |  |
| Tumor response |  |  |  |  | 0.087 |
| Partial response | 3 (4.3) | 2 (13.3) | 0 (0.0) | 1 (3.3) |  |
| Stable disease | 65 (94.2) | 13 (86.7) | 23 (95.8) | 29 (96.7) |  |
| Progressive disease | 1 (1.4) | 0 (0.0) | 1 (4.2) | 0 (0.0) |  |
